# Back to the Basics: Developing Advanced Metal–Organic Frameworks Using Fundamental Chemistry Concepts

**DOI:** 10.1021/acsnanoscienceau.2c00046

**Published:** 2022-12-27

**Authors:** Kent O. Kirlikovali, Sylvia L. Hanna, Florencia A. Son, Omar K. Farha

**Affiliations:** † Department of Chemistry and International Institute for Nanotechnology, Northwestern University, Evanston, Illinois 60208, United States; ‡ Department of Chemical and Biological Engineering, Northwestern University, Evanston, Illinois 60208, United States

**Keywords:** Metal−organic frameworks, fundamental chemistry, hard/soft acid/base theory, periodic trends, crystallization kinetics

## Abstract

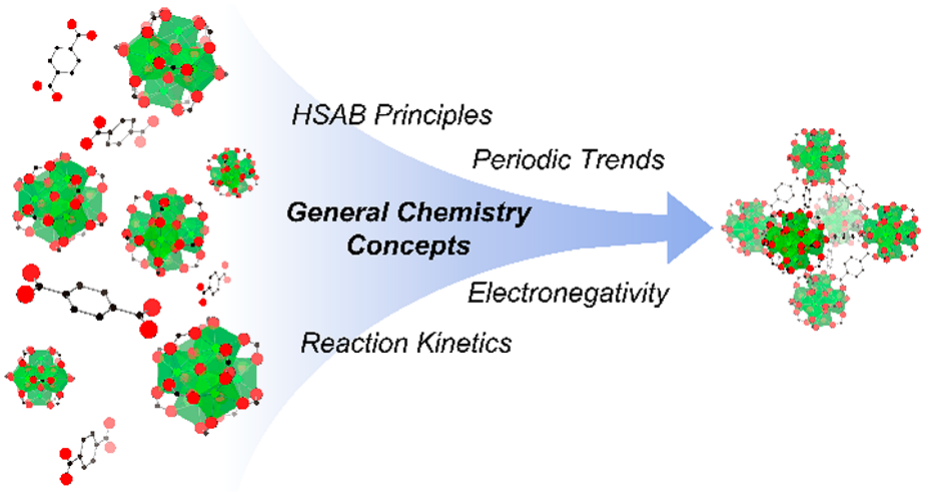

Over the past 25 years, metal–organic frameworks (MOFs) have developed into an increasingly intricate class of crystalline porous materials in which the choice of building blocks offers significant control over the physical properties of the resulting material. Despite this complexity, fundamental coordination chemistry design principles provided a strategic basis to design highly stable MOF structures. In this Perspective, we provide an overview of these design strategies and discuss how researchers leverage fundamental chemistry concepts to tune reaction parameters and synthesize highly crystalline MOFs. We then discuss these design principles in the context of several literature examples, highlighting both relevant fundamental chemistry principles and additional design principles required to access stable MOF structures. Finally, we envision how these fundamental concepts may offer access to even more advanced structures with tailored properties as the MOF field looks toward the future.

## Introduction

**Figure 1:**
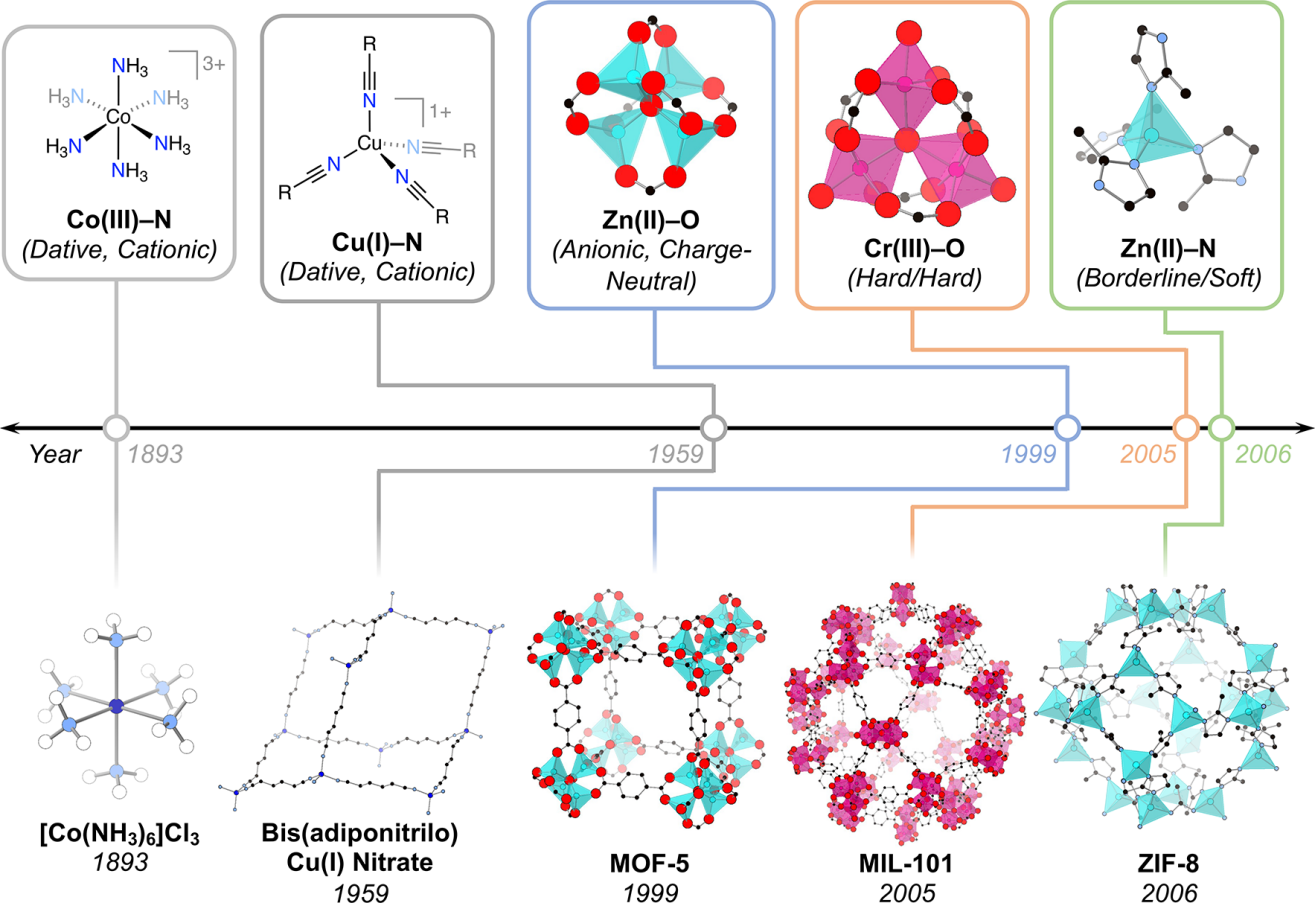
Historical evolution from initial coordination complexes to MOFs. The top panels illustrate node structures and highlight the metal–ligand bonding interactions for each material, and the bottom panels show the full structures. Black = carbon; red = oxygen; blue = nitrogen; teal = zinc; pink = chromium. Hydrogens are omitted for clarity.

Metal–organic frameworks (MOFs), which are extended periodic structures that feature inorganic nodes or clusters coordinated to multitopic organic linkers, have emerged within the last couple decades, and judicious choice of these building blocks offers access to a vast range of MOFs with targeted properties.^1^ Due to their highly tunable nature and porous, crystalline structures, MOFs have found use in a variety of applications, ranging from gas storage and separations to catalysis and chemical sensing. Despite their relatively recent development, the conceptual framework for MOF chemistry was inspired by early studies on transition-metal coordination complexes from over a century ago. For instance, Alfred Werner introduced the concepts of coordination number and coordination geometry, and he correctly proposed the structural configuration of the cobalt-ammine salt, [Co(NH_3_)_6_]Cl_3_, which includes neutral ammine ligands octahedrally coordinated to a Co(III) center (Figure 1).^2^ Initial examples of porous coordination polymers (PCPs) contained building blocks inspired by these early coordination complexes, such as neutral ligands and late-transition metal ions, but the amorphous nature of these PCPs, as well as their tendency to collapse upon attempts at solvent removal, limited their use in potential applications. To address these challenges, researchers leveraged fundamental chemistry concepts to progress from amorphous coordination polymers to crystalline MOFs with permanent porosity and, in the process, developed strategies to design and synthesize stable MOFs.

Although MOF structures have increased in complexity over time, the concepts for this evolution are rooted in principles of fundamental chemistry. In this Perspective, we highlight several prominent *general chemistry* concepts that have guided advancements in MOF chemistry. In particular, we focus on these concepts in the context of the strategic linker and node design of robust MOFs (part 1), as well as important considerations for tuning reaction parameters to synthesize the desired crystalline frameworks (part II). Finally, we discuss several literature examples that highlight specific fundamental chemistry concepts that facilitated the development of increasingly stable MOFs (part III).

## Part 1: Conceptual Node and Linker Design for Stable MOFs

### Early Coordination Networks

The earliest examples of extended inorganic coordination polymers were reminiscent of the types of coordination complexes studied by Werner.^2,3^ For example, Prussian blue^4^ and Hoffman clathrates^5^ both contain transition metal ions and cyanide ligands and form extended 3-D and 2-D framework structures, respectively. In both structures, the cyanide groups act as bridging ligands since they form dative bonds through N and anionic bonds through C. Subsequent examples of coordination networks built from these concepts and incorporated organic linkers with multiple nitrile groups coordinated to Cu(I) sites, starting with ditopic alkyl-based linkers, albeit in amorphous frameworks (bis(adiponitrilo) Cu(I) nitrate in Figure 1).^6^ Replacing the flexible alkyl groups with rigid aromatic building blocks by Robson to form tetratopic nitrile-based linkers with either a tetrahedral^7^ or a planar^8^ geometry afforded more rigid coordination networks with large porous channels; however, these frameworks were not stable to activation and removal of solvent guest molecules from the pores. Expanding beyond nitrile-based linkers, the Zaworotko^9^ and Kitagawa^10,11^ groups investigated 4,4′-bipyridine in the formation of coordination networks with transition metals such as Zn(II), Cu(II), Ni(II), and Co(II) ions.

### Permanently Porous Frameworks

**Figure 2:**
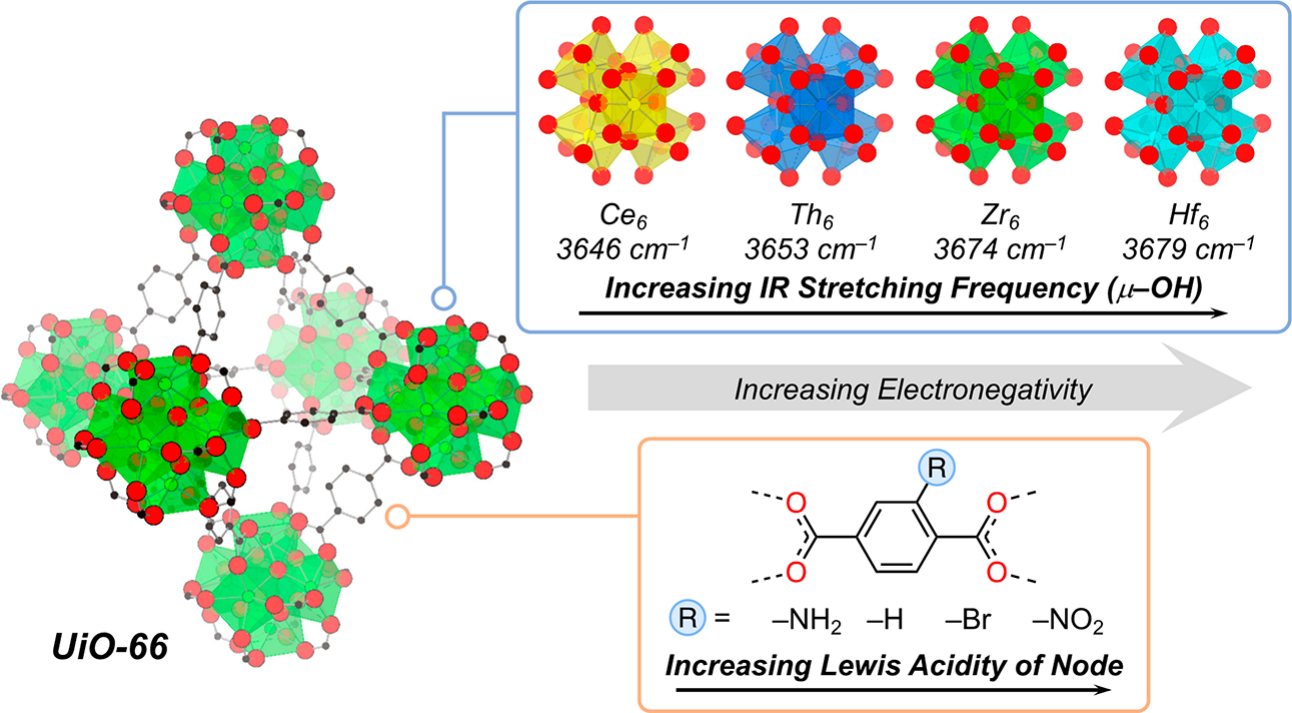
Examples of electronegativity of the metal node influences the IR stretching frequency of the node-bound hydroxyl group (top) and how electronegativity of the linker affects Lewis acidity of the Zr_6_ node (bottom).

Until this point, the selection of organic linkers was limited to neutral polynitriles or polypyridines that formed dative bonds with soft transition metal ions, and these relatively weak coordination bonds limited access to solvent-free frameworks that did not collapse upon guest removal and activation. In 1998, Yaghi and co-workers introduced a new design concept in which negatively charged carboxylate linkers coordinated to positively charged transition metal-based nodes to form charge neutral frameworks that were stable following removal of solvent guest molecules, affording the first MOFs that exhibited permanent porosity (MOF-5 in Figure 1).^12,13^ In addition, these frameworks were among the first to include transition metal-based clusters as nodes, which formed the foundation for the development of polynuclear secondary building units (SBUs) with increased complexity relative to individual transition metal ions.^3^ For instance, Férey and colleagues incorporated a high-valent Cr(III)-oxo trimer node into a framework with a ditopic carboxylate linker to generate MIL-101, which exhibited a Brunauer–Emmett–Teller (BET) area of 4100 m^2^/g and pores of about 3 nm in diameter.^14^ Moreover, MIL-101 is exceptionally stable in the presence of water owing to the strong Cr(III)–O coordination bonds that form both in the metal-oxo node and between the node and the linker (Figure 1). Importantly, these studies provided the blueprint to strategically target stable MOF structures with permanent porosity, and these advancements were grounded in fundamental chemistry concepts related to Pearson’s hard/soft acid/base (HSAB) theory.^15−17^ The carboxylate-based linkers are considered hard bases, and researchers quickly demonstrated that these linkers form robust coordination bonds with high-valent metal ions that act as hard acids, such as Ti(IV), Cr(III), Al(III), Fe(III), and Zr(IV) (e.g., UiO-66 in Figure 2).^15^ Similarly, borderline acids that are intermediate between soft and hard, such as low-valent Co(II), Ni(II), Cu(II), and Zn(II), form strong bonds with azolates and other soft bases,^15^ as exemplified by the Zn(II)- and imidazolate-based zeolitic imidazolate frameworks (ZIFs; ZIF-8 in Figure 1, also called MAF-4).^18,19^ Eddaoudi and co-workers also demonstrated that carboxylate-based linkers can afford stable MOFs when combined with metals ranging from intermediate hardness, such as the In(III)-based soc-MOF,^20^ to the harder Al-soc-MOF.^21^ Notably, MOFs comprising carboxylate bases and high-valent metal ions typically exhibit excellent stability in strongly acidic conditions and limited stability in highly basic solutions due to the low p*K*_a_ of the carboxylic acids, while MOFs with azolate bases and low-valent metal ions are highly stable in basic solutions but show limited stability in acidic solutions.^15^ These complementary stability profiles offer one example of how researchers can tailor MOF design to the desired application. With straightforward access to a wide variety of stable MOFs now available, researchers then focused on how fundamental concepts such as periodic trends and electronegativity of functional groups on the organic linkers affected the physical properties and reactivity profiles of MOFs.

### Rationally Tuning MOF Properties

Owing to the inherent tunability of MOFs, researchers could synthesize several isostructural analogues with different metals to elucidate periodic trends related to physical properties such as acidity, stability, and catalytic reactivity. For example, researchers synthesized a series of isoreticular analogues of MIL-100-M (M(III) = Sc, V, Cr, Fe, Al, In), which feature trimers of M(III)-based trinuclear clusters coordinated to trimesate linkers, and determined the relative Lewis acidity of the metal sites using infrared (IR) spectroscopy.^22,23^ Monitoring the diagnostic stretch corresponding to CD_3_CN molecules coordinated to open metal sites indicates that MIL-100-Al and -Cr feature the strongest Lewis acid sites among this series. Our group synthesized a series of MOFs based on the same trinuclear metal nodes as MIL-100 but with a trigonal prismatic carboxylate-based linker, NU-1500-M (M(III) = Sc, Cr, Fe), and found that although NU-1500-Cr is exceptionally hydrolytically stable and displays impressive water vapor uptake performance with small hysteresis for at least 20 adsorption–desorption cycles, NU-1500-Sc and -Fe are significantly less hydrolytically stable.^24^ These results emphasize how subtle differences in Lewis acidity or coordination bond strength can drastically influence the physical properties of the resulting MOF, even among series that all contain components rationally selected according to HSAB principles. In addition to IR studies using probe molecules, simple acid–base titrations can also provide insight into the Lewis acidity of metal ions in the nodes. For instance, in the triazole-based M-MFU-4l series (M = Co(II), Ni(II), Cu(II), and Zn(II)), the p*K*_a_ values of the metal-bound water molecules are 8.5, 8.3, 6.8, and 7.7, respectively, suggesting that Cu and Zn are relatively more Lewis acidic than Co and Ni.^25^ Importantly, this trend corroborates the catalytic data as the most Lewis acidic Cu(II)-MFU-4l exhibits optimal performance as a hydrolysis catalyst among this series, highlighting how studies on fundamental properties and periodic trends can inform catalyst design.

Similar to periodic trends across rows, incorporating different metals from the same group into an isostructural series of MOFs offers an avenue to study how properties such as the metal ion size and electronegativity affect the physical properties of the MOF. For example, our group previously illustrated how varying the group 4 metal in the hexanuclear node of M-UiO-66 or M-MOF-808 (M(IV) = Zr, Hf, Ce, Th) influences the IR stretch of the hydroxyl groups bound to the nodes, and we demonstrated that this stretching frequency becomes more red-shifted as the electronegativity of the metal ion decreases (Figure 2, top).^26^ Notably, this shift also correlates with the binding affinity of labile aqua ligands that are coordinated to open metal sites, which can inform catalyst design in systems where these nodes serve as active sites in Lewis acid-catalyzed reactions. In addition to modifying the electronic properties of the node-based active sites by changing the identity of the metal ion, varying the electronic nature of the functional group on the organic linker can also greatly influence active site reactivity. In one example, introducing increasingly electron-withdrawing functional groups onto the linker of UiO-66 enhanced the Lewis acidity of the Zr-based active sites and subsequently resulted in faster reaction rates when UiO-66 was used as a Lewis acid catalyst (Figure 2, bottom).^27^ Combined, these studies illustrate how leveraging fundamental concepts, such as periodic trends, Lewis acidity, and electronegativity, in the context of MOF node and linker choice offers several straightforward strategies to predictably modulate the physical properties of MOFs.

## Part 2: Fundamental Chemistry Considerations for Reaction Parameters

Fundamental chemistry principles not only frame initial MOF linker and node design, but also guide the choice of reaction conditions to enable successful MOF synthesis. Standard MOF reaction conditions most commonly involve solvothermal syntheses in organic media at elevated temperatures. In this section, we outline considerations for selected reaction constituents (linker, node, modulator, solvent), reaction parameters (concentration, temperature, time), and reaction progress (nucleation, growth), and we emphasize the underlying chemistry tenets behind them.

### Linker and Node

MOF synthesis begins with the linker and node building blocks, the directionality and connectivity of which define the resulting MOF topology, structure, and ultimately properties. Linking these building blocks together to form the desired MOF product relies on the fundamental chemistry concepts of reactant stoichiometry and limiting and excess reagent. For example, the Zhou group observed the formation of different MOFs depending on the molar ratio of the anthrancene-9,10-dicarboxylic acid linker and the Zn SBU employed in the reaction. Higher metal to ligand ratios produced PCN-13, while systematically decreasing the metal to ligand molar ratio generated the additional products PCN-132 and PCN-131.^28^ Thus, adjustments must be performed to determine optimal initial molar ratios, with certain building blocks acting as limiting or excess reagents.

### Modulator

While the linker and node starting components define the framework architecture, combining these building blocks alone commonly results in fast precipitation and low crystallinity. Researchers discovered that an added modulator would slow down self-assembly and encourage MOF organization into a crystalline framework. Modulators also influence MOF traits such as particle size, phase purity, and defects, and most can be classified as either coordinating or Brønsted acid modulators.^29^ Fundamental chemistry concepts such as modulator stoichiometry, HSAB binding chemistry, conjugate base strength, and p*K*_a_ influence reaction modulation to achieve desired crystallization, as detailed below.

**Figure 3:**
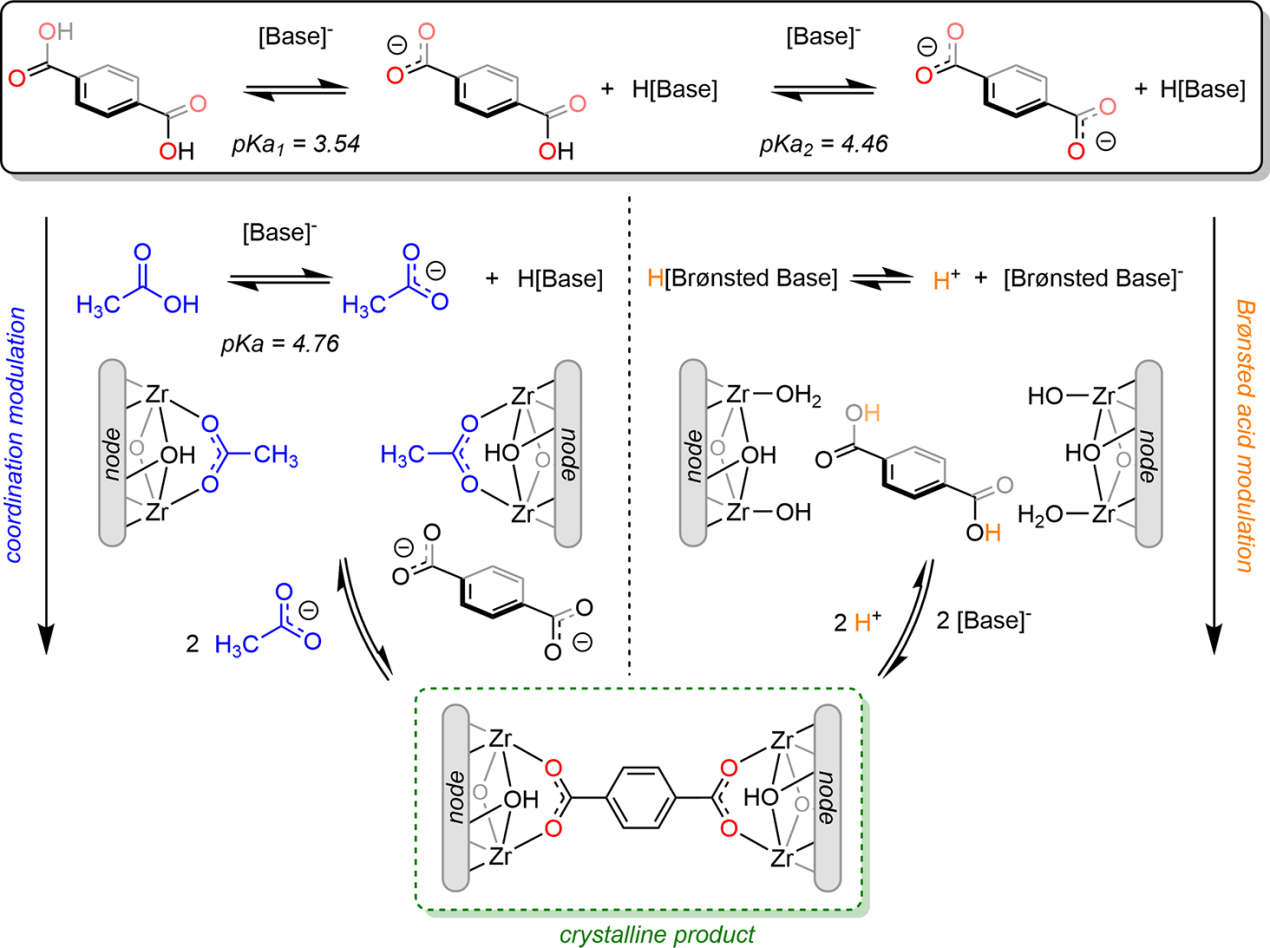
Modulator role in MOF synthesis. Linker equilibrium (top), coordination modulation (left), Brønsted acid modulation (right), and final linker coordination to node sites (bottom).

Coordinating modulators are monotopic acid moieties added in molar excess with similar binding chemistry to the linker. For example, carboxylate modulators are often selected for systems with carboxylate-binding linkers, and phosphonate modulators for systems with phosphonate-binding linkers. Under deprotonation conditions (discussed below), both the linker (Figure 3, top) and coordinating modulator (Figure 3, left) convert to their corresponding conjugate bases which then compete for binding sites on metal ions. Since coordinating modulators are monotopic, they may bind to one metal ion (Figure 3, left), but they cannot bridge to a second metal ion to form a connected framework. High temperature conditions increase the lability of linker and modulator which allows for substitution of monotopic modulators with multitopic linkers that can bridge metal ions together (Figure 3, bottom). This resulting linker competition with excess modulator slows down the kinetics of self-assembly and thereby increases crystallinity.

In addition to aligned hard/hard or soft/soft binding chemistry, compatible competition between coordinating modulator and linker depends on their relative p*K*_a_ values. A desired modulator should become deprotonated at a similar p*K*_a_ to the linker in order for both conjugate bases to simultaneously compete for metal binding sites. For example, in the reported synthesis of UiO-66, acetic acid (p*K*_a_ = 4.76, Figure 3, left) is chosen over modulators like trifluoroacetic acid (p*K*_a_ = 0.23) or stearic acid (p*K*_a_ = 10.15) as it is more compatible with the terephthalic acid linker with p*K*_a__1_ = 3.54 and p*K*_a__2_ = 4.46 (Figure 3, top).^26^ In this way, both the modulator identity and the molar ratio can both be controlled to achieve the desired crystallization.

Brønsted acid modulators also decrease the kinetics of MOF crystallization. These modulators include acids like HCl or HBF_4_.^30^ Instead of slowing down binding of the linker’s conjugate base by blocking metals sites, Bronsted acid modulators slow down MOF self-assembly by protonating the linker and preventing its conjugate base from being favored (Figure 3, right). Thus, strong Brønsted acids with weak conjugate bases provide a higher concentration of available H^+^ ions to modulate linker protonation.

### Solvent

In a conventional MOF synthesis, the solvent initially behaves as a reaction medium which solubilizes the linker, node, modulator, and any other reaction components. Additionally, to prevent evaporation, solvents should possess boiling points higher than the selected synthesis temperature. Thus, to account for the basic chemistry considerations of solubility and boiling point, formamide-based solvents such as *N*,*N*-dimethylformamide (DMF) and *N*,*N*-diethylformamide (DEF) commonly appear in reported MOF syntheses.

Solvent may also participate as a key contributor in driving the reaction forward and/or favoring certain products due to basic chemistry concepts such as conjugate base strength and steric hindrance. For example, at higher temperatures, DMF and DEF decompose to their corresponding basic amines (dimethylamine and diethylamine, respectively).^29^ These bases then initiate the self-assembly process by deprotonating the linker and coordinating modulator. Slow decomposition of DMF and DEF also helps prevent the amorphous precipitation of product. Finally, solvent size can favor specific MOF products due to templating effects from steric hindrance. For example, Ma et al. reported how the use of DMF as solvent produced an interpenetrated MOF with smaller pores, while switching the solvent to the bulkier DEF resulted in the noninterpenetrated analog with larger pores.^31^

### Enthalpy of Formation and Reaction Temperature

MOF formation depends not only on the appropriate linker, node, modulator, and solvent choice, but also on the reaction conditions selected. Fundamental chemistry concepts including HSAB theory and kinetic energy help explain enthalpies of MOF formation and the use of higher temperatures for MOF reactions.

Using experimental calorimetry studies, the Navrotsky group has observed both endothermic and exothermic enthalpies of MOF formation relative to the dense phases of the reactant constituents. For example, endothermic enthalpies of formation were reported for MOF-5,^32^ Mg-MOF-74, and Ni-MOF-74.^33^ Conversely, stabilizing solvent-framework interactions in Zn-HKUST-DMF and Cu-HKUST-H_2_O resulted in exothermic enthalpies of formation.^34^ Likewise, studies in ZIF systems demonstrated that stabilizing changes in local Zn coordination environment from Zn–O (starting material with less favorable borderline-hard pairing) to Zn–N (MOF with more favorable borderline-soft pairing) drove ZIF formation and promoted exothermic enthalpies of formation.^35^

For MOFs with both endothermic and exothermic enthalpies of formation, added temperature raises the kinetic energy of reaction constituents to be equal to or greater than the reaction’s activation energy. This in turn increases collisions between reactants, kinetically decreases the time it takes to form product, and thermodynamically favors more stable products. Additionally, high reactant kinetic energy favors modulator lability, encouraging competitive coordination. Finally, temperature affects reactant solubility and solution supersaturation, which is necessary for nucleation.^36^

### Reaction Time and Concentration

In addition to reaction temperature, the resulting MOF product can be affected by the reaction time and concentration.^37^ Kinetically favored products may appear at shorter time scales. Additionally, collision theory postulates that the reaction rate is proportional to the rate of reactant collisions. Thus, higher reaction concentration also affects nucleation of new MOF particles.

### MOF Nucleation and Growth

While tuning of reaction constituents and parameters enables certain control over MOF synthesis, MOF crystallization is a complex phenomenon which has been described by various models and hypotheses. Generally, crystallization occurs under nucleation and growth regimes. We discuss these processes and the fundamental chemistry behind them separately below, but we note that these regimes may temporally overlap.

#### MOF Nucleation

Classical nucleation theory (CNT) posits that monomeric units assemble into nuclei which remain in equilibrium with their surroundings. Monomers can be added or removed until the nuclei reach a critical size where growth then occurs. Supersaturation drives this process, and since temperature affects the degree of supersaturation, nucleation depends on a critical concentration and temperature. Once nucleation occurs, it gives rise to a free energy change in the system.^38^

While CNT provides a basis for how concentration and temperature control favors nucleation, alternative hypotheses have been proposed to better model MOF nucleation. Several models suggest that units composed of either single monomers (linker and metal ions), portions of SBUs, or full SBUs act as the building units which assemble into nuclei.^36^ Recently, Liu et al. proposed MOF nucleation of ZIF-8 through a nonclassical pathway composed of phase separation, condensation, and crystallization.^39^ Although various models exist, nucleation in principle reduces the level of supersaturation of the solution and promotes the particle growth regime.

#### MOF Growth

Unlike classical materials, MOF growth is not only directed by the geometry of its building blocks, but also by their coordinating directionality. Classical growth theories indicate that crystal growth is a thermodynamically favorable event under supersaturation conditions. Monomeric species diffuse from the solution to the crystal surface and are then adsorbed or incorporated onto the surface. Other theories such as Ostwald’s Rule of Stages suggest multiple sequential precipitations to form a fully grown crystallite.^36^ While multiple modes of growth may occur simultaneously, a recent report by Han et al. uses dark field microscopy to shed light on the kinetic parameters of MOF crystal growth. The authors showed that increasing reaction temperature raises MOF growth rates. However, reaction orders corresponding to the linker and node reveal that these building blocks influence MOF growth independent of the ratio of metal and linker within the MOF chemical formula. This suggests that linker and node SBUs are not correlated during MOF growth. Additionally, the authors showed evidence for a dynamic transition layer at the interface between MOF crystal bulk and solution where intermediate ordering is present.^40^ Thus, both temperature and reactant concentration are determining factors for MOF growth. Other factors such as modulator, capping agent, and solvent can control crystal size, morphology, and polymorphism during the growth process.

## Part 3: Developing Increasingly Stable MOFs

Over the past few decades, researchers have leveraged their knowledge of chemistry to develop more robust MOFs. Advances in coordination chemistry and synthetic strategies have allowed the community to attain architecturally, chemically, thermally, and hydrolytically stable materials. Here, we highlight a few examples from the literature to demonstrate how the aforementioned chemistry principles are applied to enhance the thermodynamic and kinetic stability of MOFs.

### Architecturally Stable MOFs with High Porosity

**Figure 4:**
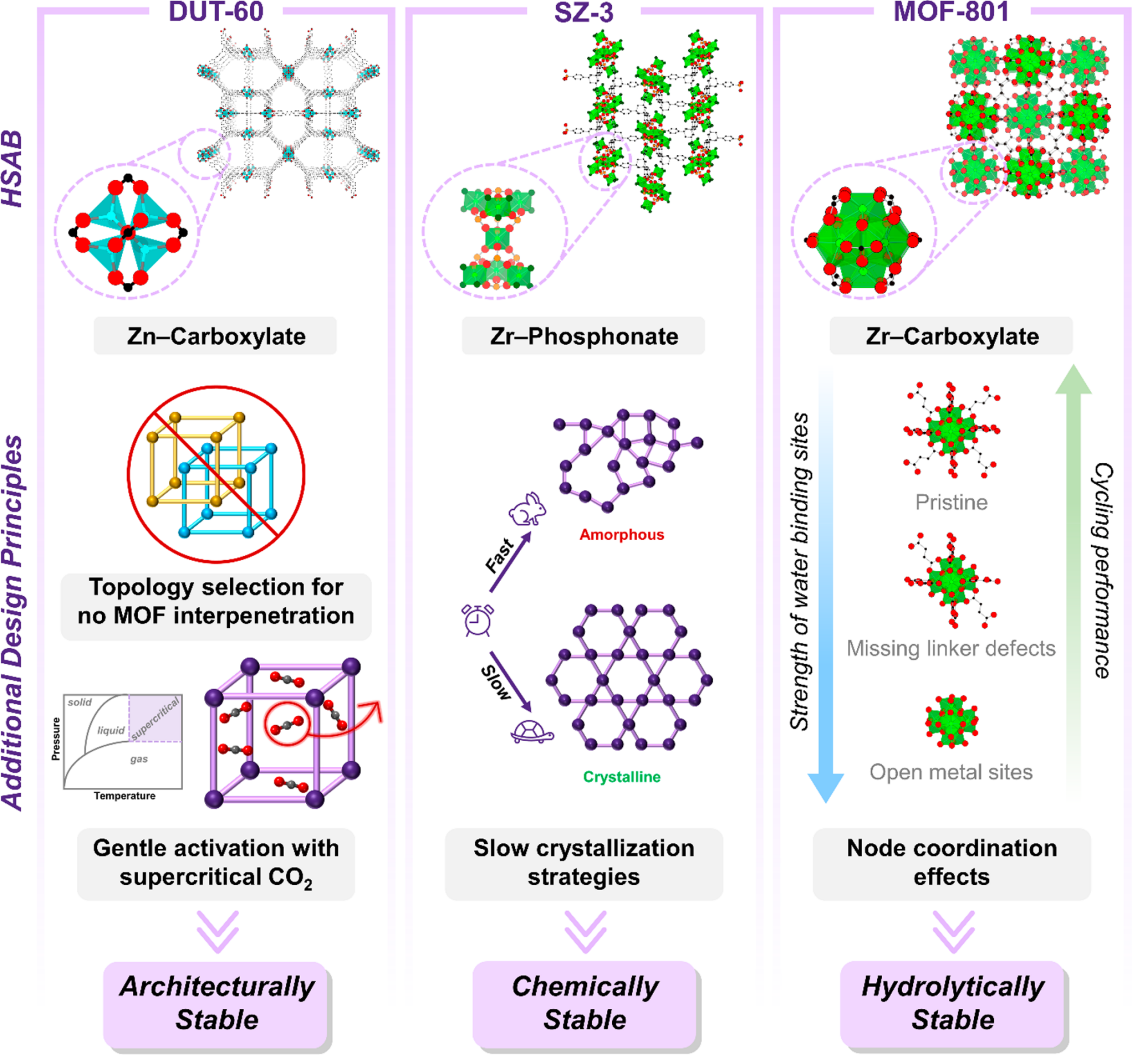
Visualization of chemistry principles in DUT-60 (left), SZ-3 (middle), and MOF-808 (right). Black = carbon; red = oxygen; teal = zinc; green = zirconium; dark green = fluorine; orange = phosphorus. Hydrogens are omitted for clarity.

Accessing high porosity MOFs is critical for applications such as gas storage. While the theoretical surface area limit for porous materials has been proposed to be ∼10500 m^2^/g based on poly(*p*-phenylene), or ∼14600 m^2^/g based on ethynyl units,^41^ the realization of such surface areas requires meticulous framework design. Oftentimes, high surface areas can be achieved via linker elongation; however, this must be coupled with the selection of robust topologies, strong metal–ligand coordination, and careful activation strategies to prevent the collapse of the framework (Figure 4, left panel). In 2018, Kaskel and co-workers developed a mesoporous MOF named DUT-60 with **ith-d** topology, which has the highest recorded accessible pore volume (5.02 cm^3^/g).^42^ As this structure consists of Zn_4_O^6+^ clusters, in which Zn(II) is a borderline Lewis acid, and tritopic carboxylate-based linkers, which are hard bases, additional design principles had to be applied to afford the final structure architectural stability. Notably, the incorporation of auxiliary ligands—additional ligands integrated into the MOF framework—cross-linked the larger pores with smaller ones, providing structural stability and preventing MOF interpenetration, which would decrease the overall porosity. The ditopic carboxylate-based auxiliary ligands imparted the necessary architectural stability to prevent pore collapse during solvent removal. Finally, activation using supercritical CO_2_, which eliminates the impact of surface tension present in thermal activations,^43^ allowed Kaskel and colleagues to attain a BET area of 7840 m^2^/g for DUT-60 when using two of the four BET consistency criteria, fitting parameters which increase the accuracy of BET areas extracted from experimental isotherms.^44^ Within the Farha group, we have also employed similar strategies to synthesize NU-1501-Al. The use of a hard Al(III) Lewis acid and hard carboxylate-based base, incorporation of a rigid linker, selection of **acs** topology, and activation via supercritical CO_2_ results in a BET area of 7310 m^2^/g when satisfying all four BET consistency criteria. When fulfilling the first two BET consistency criteria, such as for DUT-60, the apparent BET area is estimated to reach 9150 m^2^ g^–1^.^45^

### Chemically Robust MOFs

Much of MOF stability can be attributed to the fundamental chemistry concept of Pearson’s HSAB. Compared to the combination of tetravalent zirconium, a hard Lewis acid, and hard carboxylate ligands, even stronger coordination strength can be reached when using phosphonates, which possess higher p*K*_a_ values, in line with the HSAB principle. Despite the potential for chemically robust Zr-phosphonate MOFs, due to the strong affinity of Zr^4+^ and phosphonate linkers, crystalline structures can be difficult to attain owing to the rapid precipitation of amorphous products. Therefore, additional design rules for crystallization kinetics must be applied to target crystalline MOFs. Wang and co-workers were able to circumvent this issue by using an ionothermal synthesis to prevent the hydrolysis of Zr(IV), slow down crystallization kinetics, and thereby fabricate three Zr-phosphonate MOFs: SZ-1, SZ-2, and SZ-3 (Figure 4, middle panel).^46^ In this strategy, an ionic liquid is used as both the solvent and the structure-directing agent, or template.^47^ Notably, by taking advantage of HSAB principles, they designed the first MOF to survive in aqua regia.

Beyond hard acid/hard base MOFs, high chemical stability has also been observed in azolate-based frameworks composed of borderline acids and bases, such as with ZIFs containing divalent transition metals.^18^ In the examples of these imidazolate-based MOFs, the hydrophobic pore environments aid in repelling water and combination of the borderline Lewis acids with the N-donor ligands results in strong coordination. Interestingly, other azolate-based MOFs, such as Fe_2_(bdp)_3_ (bdp^2–^ = 4,4′-(1,4-phenylene)bis(pyrazolate))—which combines a hard acid, Fe(III), with a borderline base—have also been illustrated to possess high chemical stability,^48^ indicating that linker identity can be used to increase MOF stability when HSAB principles are not fully leveraged.

### Hydrolytically Stable MOFs

Finally, we conclude this section by discussing MOFs with hydrolytic stability, generally requisite for the practical implementation of these materials. In the presence of water, MOFs can degrade due to hydrolysis or linker displacement. As such, researchers can implement strategies to mollify the impact of water by tuning the hydrophobicity of the organic linkers or altering the secondary building unit. In a comprehensive study conducted in 2014, Yaghi and co-workers probed 20 MOFs for water adsorption, including 5 new Zr_6_O_4_(OH)_4_(−CO_2_)_*n*_-based materials where *n* = 6, 8, 10, or 12.^49^ The Zr-carboxylate coordination strength aided in enhancing the hydrolytic stability of these materials, as evidenced by their cycling performance (Figure 4, right panel). However, not all Zr-carboxylate MOFs performed well, indicating that solely focusing on HSAB principles is not enough to develop a robust framework and one must also consider the kinetic stability of MOFs (e.g., node connectivity). Several materials—such as MOF-805, MOF-806, and MOF-808—experienced a loss of water uptake capacity upon cycling experiments, along with a loss of porosity, indicative of partial framework collapse. Likewise, MOF-804, DUT-67, MOF-74, and CAU-6 had a significant disparity in uptake from the first and second cycle. In these examples, having hydrophilic frameworks and/or strong adsorption sites for water was disadvantageous for maintaining water adsorption capacity either due to MOF degradation or energetically demanding regeneration requirements. Similar to the Chemically Robust MOFs section, hydrolytic stability can also be acquired by using hydrophobic linkers, such as with ZIFs.^18^

## Conclusions and Outlook

By drawing from fundamental chemistry principles, we can more deeply understand and design MOF materials. We hope that by outlining these underlying concepts, we can aid MOF researchers—both new and experienced—in their investigations into MOF crystallization as they engineer highly robust MOFs. This overview of MOF design and growth enables us to push the boundaries of coordination chemistry moving forward. Looking forward, we anticipate that the application of fundamental chemistry concepts will aid in addressing challenges MOFs face in fields such as hydrogen storage, water capture from arid conditions, or enzyme encapsulation. For instance, with hydrogen storage, how can we continue to apply HSAB principles to design robust materials with low-valent metal sites to enhance hydrogen-framework interactions? When considering MOFs that need to undergo thousands of water adsorption–desorption cycles, how can we ensure the hydrolytic and architectural stability of the framework? Can we design highly porous MOFs that enable the encapsulation of large enzymes, without the use of auxiliary ligands? Finally, beyond focusing on applications, we encourage scientists to continue studying the fundamentals of MOF chemistry, such as MOF growth, through techniques such as isothermal titration chemistry, which provides insights into thermodynamic parameters, or high-resolution transmission electron microscopy. As this field looks toward the future, we envision that innovative scientists will address these posed questions and even greater challenges by expanding upon new general chemistry principles.

## References

[ref1] Kaskel S. The Chemistry of Metal–Organic Frameworks: Synthesis, Characterization, and Applications; Wiley-VCH Verlag GmbH & Co. KGaA, 2016; Vol. 1, pp 1–849.

[ref2] Werner A. Beitrag zur Konstitution anorganischer Verbindungen. Z. Anorg. Allgem. Chem. 1893, 3 (1), 267–330. 10.1002/zaac.18930030136.

[ref3] Chen Z.; Wasson M. C.; Drout R. J.; Robison L.; Idrees K. B.; Knapp J. G.; Son F. A.; Zhang X.; Hierse W.; Kühn C.; Marx S.; Hernandez B.; Farha O. K. The state of the field: from inception to commercialization of metal–organic frameworks. Faraday Discuss. 2021, 225 (0), 9–69. 10.1039/D0FD00103A. 33242050

[ref4] Keggin J. F.; Miles F. D. Structures and Formulæ of the Prussian Blues and Related Compounds. Nature 1936, 137 (3466), 577–578. 10.1038/137577a0.

[ref5] Powell H. M.; Rayner J. H. Clathrate Compound Formed by Benzene with an Ammonia–Nickel Cyanide Complex. Nature 1949, 163 (4145), 566–567. 10.1038/163566a0.

[ref6] Kinoshita Y.; Matsubara I.; Higuchi T.; Saito Y. The Crystal Structure of Bis(adiponitrilo)copper(I) Nitrate. Bull. Chem. Soc. Jpn. 1959, 32 (11), 1221–1226. 10.1246/bcsj.32.1221.

[ref7] Hoskins B. F.; Robson R. Infinite polymeric frameworks consisting of three dimensionally linked rod-like segments. J. Am. Chem. Soc. 1989, 111 (15), 5962–5964. 10.1021/ja00197a079.

[ref8] Abrahams B. F.; Hoskins B. F.; Michail D. M.; Robson R. Assembly of porphyrin building blocks into network structures with large channels. Nature 1994, 369 (6483), 727–729. 10.1038/369727a0.

[ref9] Subramanian S.; Zaworotko M. J. Porous Solids by Design: [Zn(4,4′-bpy)2(SiF6)]n·xDMF, a Single Framework Octahedral Coordination Polymer with Large Square Channels. Angew. Chem., Int. Ed. Engl. 1995, 34 (19), 2127–2129. 10.1002/anie.199521271.

[ref10] Noro S.-i.; Kitagawa S.; Kondo M.; Seki K. A New, Methane Adsorbent, Porous Coordination Polymer [{CuSiF6(4,4′-bipyridine)2}n]. Angew. Chem. Int. Ed 2000, 39 (12), 2081–2084. 10.1002/1521-3773(20000616)39:12<2081::AID-ANIE2081>3.0.CO;2-A. 10941021

[ref11] Kondo M.; Yoshitomi T.; Matsuzaka H.; Kitagawa S.; Seki K. Three-Dimensional Framework with Channeling Cavities for Small Molecules: {[M2(4, 4′-bpy)3(NO3)4]·xH2O}n (M = Co, Ni, Zn). Angew. Chem., Int. Ed. Engl. 1997, 36 (16), 1725–1727. 10.1002/anie.199717251.

[ref12] Li H.; Eddaoudi M.; Groy T. L.; Yaghi O. M. Establishing Microporosity in Open Metal–Organic Frameworks: Gas Sorption Isotherms for Zn(BDC) (BDC = 1,4-Benzenedicarboxylate). J. Am. Chem. Soc. 1998, 120 (33), 8571–8572. 10.1021/ja981669x.

[ref13] Li H.; Eddaoudi M.; O’Keeffe M.; Yaghi O. M. Design and synthesis of an exceptionally stable and highly porous metal-organic framework. Nature 1999, 402 (6759), 276–279. 10.1038/46248.

[ref14] Férey G.; Mellot-Draznieks C.; Serre C.; Millange F.; Dutour J.; Surblé S.; Margiolaki I. A Chromium Terephthalate-Based Solid with Unusually Large Pore Volumes and Surface Area. Science 2005, 309 (5743), 2040–2042. 10.1126/science.1116275. 16179475

[ref15] Yuan S.; Feng L.; Wang K.; Pang J.; Bosch M.; Lollar C.; Sun Y.; Qin J.; Yang X.; Zhang P.; Wang Q.; Zou L.; Zhang Y.; Zhang L.; Fang Y.; Li J.; Zhou H.-C. Stable Metal–Organic Frameworks: Design, Synthesis, and Applications. Adv. Mater. 2018, 30 (37), 1704303 10.1002/adma.201704303. 29430732

[ref16] Hamisu A. M.; Ariffin A.; Wibowo A. C. Cation exchange in metal-organic frameworks (MOFs): The hard-soft acid-base (HSAB) principle appraisal. Inorg. Chim. Acta 2020, 511, 119801 10.1016/j.ica.2020.119801.

[ref17] Pearson R. G. Hard and Soft Acids and Bases. J. Am. Chem. Soc. 1963, 85 (22), 3533–3539. 10.1021/ja00905a001.

[ref18] Park K. S.; Ni Z.; Côté A. P.; Choi J. Y.; Huang R.; Uribe-Romo F. J.; Chae H. K.; O’Keeffe M.; Yaghi O. M. Exceptional chemical and thermal stability of zeolitic imidazolate frameworks. Proc. Natl. Acad. Sci. U.S.A 2006, 103 (27), 10186–10191. 10.1073/pnas.0602439103. 16798880 PMC1502432

[ref19] Huang X.-C.; Lin Y.-Y.; Zhang J.-P.; Chen X.-M. Ligand-Directed Strategy for Zeolite-Type Metal–Organic Frameworks: Zinc(II) Imidazolates with Unusual Zeolitic Topologies. Angew. Chem. Int. Ed 2006, 45 (10), 1557–1559. 10.1002/anie.200503778. 16440383

[ref20] Liu Y.; Eubank J. F.; Cairns A. J.; Eckert J.; Kravtsov V. C.; Luebke R.; Eddaoudi M. Assembly of Metal–Organic Frameworks (MOFs) Based on Indium-Trimer Building Blocks: A Porous MOF with soc Topology and High Hydrogen Storage. Angew. Chem. Int. Ed 2007, 46 (18), 3278–3283. 10.1002/anie.200604306. 17385775

[ref21] Alezi D.; Belmabkhout Y.; Suyetin M.; Bhatt P. M.; Weseliński Ł. J.; Solovyeva V.; Adil K.; Spanopoulos I.; Trikalitis P. N.; Emwas A.-H.; Eddaoudi M. MOF Crystal Chemistry Paving the Way to Gas Storage Needs: Aluminum-Based soc-MOF for CH4, O2, and CO2 Storage. J. Am. Chem. Soc. 2015, 137 (41), 13308–13318. 10.1021/jacs.5b07053. 26364990 PMC4616230

[ref22] Horcajada P.; Surblé S.; Serre C.; Hong D.-Y.; Seo Y.-K.; Chang J.-S.; Grenèche J.-M.; Margiolaki I.; Férey G. Synthesis and catalytic properties of MIL-100(Fe), an iron(iii) carboxylate with large pores. Chem. Commun. 2007, 27, 2820–2822. 10.1039/B704325B. 17609787

[ref23] Gómez-Pozuelo G.; Cabello C. P.; Opanasenko M.; Horáček M.; Čejka J. Superior Activity of Isomorphously Substituted MOFs with MIL-100(M = Al, Cr, Fe, In, Sc, V) Structure in the Prins Reaction: Impact of Metal Type. ChemPlusChem. 2017, 82 (1), 152–159. 10.1002/cplu.201600456. 31961502

[ref24] Chen Z.; Li P.; Zhang X.; Li P.; Wasson M. C.; Islamoglu T.; Stoddart J. F.; Farha O. K. Reticular Access to Highly Porous acs-MOFs with Rigid Trigonal Prismatic Linkers for Water Sorption. J. Am. Chem. Soc. 2019, 141 (7), 2900–2905. 10.1021/jacs.8b13710. 30735359

[ref25] Mian M. R.; Chen H.; Cao R.; Kirlikovali K. O.; Snurr R. Q.; Islamoglu T.; Farha O. K. Insights into Catalytic Hydrolysis of Organophosphonates at M–OH Sites of Azolate-Based Metal Organic Frameworks. J. Am. Chem. Soc. 2021, 143 (26), 9893–9900. 10.1021/jacs.1c03901. 34160219

[ref26] Islamoglu T.; Ray D.; Li P.; Majewski M. B.; Akpinar I.; Zhang X.; Cramer C. J.; Gagliardi L.; Farha O. K. From Transition Metals to Lanthanides to Actinides: Metal-Mediated Tuning of Electronic Properties of Isostructural Metal–Organic Frameworks. Inorg. Chem. 2018, 57 (21), 13246–13251. 10.1021/acs.inorgchem.8b01748. 30299939

[ref27] Vermoortele F.; Vandichel M.; Van de Voorde B.; Ameloot R.; Waroquier M.; Van Speybroeck V.; De Vos D. E. Electronic Effects of Linker Substitution on Lewis Acid Catalysis with Metal–Organic Frameworks. Angew. Chem. Int. Ed 2012, 51 (20), 4887–4890. 10.1002/anie.201108565. 22488675

[ref28] Gao Q.; Xie Y.-B.; Li J.-R.; Yuan D.-Q.; Yakovenko A. A.; Sun J.-H.; Zhou H.-C. Tuning the Formations of Metal–Organic Frameworks by Modification of Ratio of Reactant, Acidity of Reaction System, and Use of a Secondary Ligand. Cryst. Growth Des. 2012, 12 (1), 281–288. 10.1021/cg201059d.

[ref29] Forgan R. S. Modulated self-assembly of metal–organic frameworks. Chem. Sci. 2020, 11 (18), 4546–4562. 10.1039/D0SC01356K. 34122913 PMC8159241

[ref30] Farha O. K.; Özgür Yazaydın A.; Eryazici I.; Malliakas C. D.; Hauser B. G.; Kanatzidis M. G.; Nguyen S. T.; Snurr R. Q.; Hupp J. T. De novo synthesis of a metal–organic framework material featuring ultrahigh surface area and gas storage capacities. Nat. Chem. 2010, 2 (11), 944–948. 10.1038/nchem.834. 20966950

[ref31] Ma L.; Lin W. Chirality-Controlled and Solvent-Templated Catenation Isomerism in Metal–Organic Frameworks. J. Am. Chem. Soc. 2008, 130 (42), 13834–13835. 10.1021/ja804944r. 18823117

[ref32] Hughes J. T.; Navrotsky A. MOF-5: Enthalpy of Formation and Energy Landscape of Porous Materials. J. Am. Chem. Soc. 2011, 133 (24), 9184–9187. 10.1021/ja202132h. 21598944

[ref33] Voskanyan A. A.; Goncharov V. G.; Novendra N.; Guo X.; Navrotsky A. Thermodynamics Drives the Stability of the MOF-74 Family in Water. ACS Omega 2020, 5 (22), 13158–13163. 10.1021/acsomega.0c01189. 32548502 PMC7288594

[ref34] Bhunia M. K.; Hughes J. T.; Fettinger J. C.; Navrotsky A. Thermochemistry of Paddle Wheel MOFs: Cu-HKUST-1 and Zn-HKUST-1. Langmuir 2013, 29 (25), 8140–8145. 10.1021/la4012839. 23724924

[ref35] Novendra N.; Marrett J. M.; Katsenis A. D.; Titi H. M.; Arhangelskis M.; Friščić T.; Navrotsky A. Linker Substituents Control the Thermodynamic Stability in Metal–Organic Frameworks. J. Am. Chem. Soc. 2020, 142 (52), 21720–21729. 10.1021/jacs.0c09284. 33326738

[ref36] Van Vleet M. J.; Weng T.; Li X.; Schmidt J. R. In Situ, Time-Resolved, and Mechanistic Studies of Metal–Organic Framework Nucleation and Growth. Chem. Rev. 2018, 118 (7), 3681–3721. 10.1021/acs.chemrev.7b00582. 29514005

[ref37] Cheetham A. K.; Kieslich G.; Yeung H. H. M. Thermodynamic and Kinetic Effects in the Crystallization of Metal–Organic Frameworks. Acc. Chem. Res. 2018, 51 (3), 659–667. 10.1021/acs.accounts.7b00497. 29451770

[ref38] Bruckner E. P.; Stupp S. I. Designing supramolecular polymers with nucleation and growth processes. Polym. Int. 2022, 71 (5), 590–595. 10.1002/pi.6384.

[ref39] Liu X.; Chee S. W.; Raj S.; Sawczyk M.; Král P.; Mirsaidov U. Three-step nucleation of metal–organic framework nanocrystals. Proc. Natl. Acad. Sci. U.S.A 2021, 118 (10), e2008880118 10.1073/pnas.2008880118. 33649204 PMC7958460

[ref40] Han J.; He X.; Liu J.; Ming R.; Lin M.; Li H.; Zhou X.; Deng H. Determining factors in the growth of MOF single crystals unveiled by in situ interface imaging. Chem. 2022, 8 (6), 1637–1657. 10.1016/j.chempr.2022.03.006.

[ref41] Farha O. K.; Eryazici I.; Jeong N. C.; Hauser B. G.; Wilmer C. E.; Sarjeant A. A.; Snurr R. Q.; Nguyen S. T.; Yazaydın A. Ö.; Hupp J. T. Metal–Organic Framework Materials with Ultrahigh Surface Areas: Is the Sky the Limit?. J. Am. Chem. Soc. 2012, 134 (36), 15016–15021. 10.1021/ja3055639. 22906112

[ref42] Hönicke I. M.; Senkovska I.; Bon V.; Baburin I. A.; Bönisch N.; Raschke S.; Evans J. D.; Kaskel S. Balancing mechanical stability and ultrahigh porosity in crystalline framework materials. Angew. Chem. Int. Ed 2018, 57 (42), 13780–13783. 10.1002/anie.201808240. 30160076

[ref43] Nelson A. P.; Farha O. K.; Mulfort K. L.; Hupp J. T. Supercritical processing as a route to high internal surface areas and permanent microporosity in metal–organic framework materials. J. Am. Chem. Soc. 2009, 131 (2), 458–460. 10.1021/ja808853q. 19108683

[ref44] Gómez-Gualdrón D. A.; Moghadam P. Z.; Hupp J. T.; Farha O. K.; Snurr R. Q. Application of Consistency Criteria To Calculate BET Areas of Micro- And Mesoporous Metal–Organic Frameworks. J. Am. Chem. Soc. 2016, 138 (1), 215–224. 10.1021/jacs.5b10266. 26651496

[ref45] Chen Z.; Li P.; Anderson R.; Wang X.; Zhang X.; Robison L.; Redfern L. R.; Moribe S.; Islamoglu T.; Gómez-Gualdrón D. A.; Yildirim T.; Stoddart J. F.; Farha O. K. Balancing volumetric and gravimetric uptake in highly porous materials for clean energy. Science 2020, 368 (6488), 297–303. 10.1126/science.aaz8881. 32299950

[ref46] Zheng T.; Yang Z.; Gui D.; Liu Z.; Wang X.; Dai X.; Liu S.; Zhang L.; Gao Y.; Chen L.; Sheng D.; Wang Y.; Diwu J.; Wang J.; Zhou R.; Chai Z.; Albrecht-Schmitt T. E.; Wang S. Overcoming the crystallization and designability issues in the ultrastable zirconium phosphonate framework system. Nat. Commun. 2017, 8 (1), 15369 10.1038/ncomms15369. 28555656 PMC5459948

[ref47] Parnham E. R.; Morris R. E. Ionothermal Synthesis of Zeolites, Metal–Organic Frameworks, and Inorganic–Organic Hybrids. Acc. Chem. Res. 2007, 40 (10), 1005–1013. 10.1021/ar700025k. 17580979

[ref48] Wang Z.; Bilegsaikhan A.; Jerozal R. T.; Pitt T. A.; Milner P. J. Evaluating the Robustness of Metal–Organic Frameworks for Synthetic Chemistry. ACS Appl. Mater. Interfaces 2021, 13 (15), 17517–17531. 10.1021/acsami.1c01329. 33822586 PMC8232555

[ref49] Furukawa H.; Gándara F.; Zhang Y.-B.; Jiang J.; Queen W. L.; Hudson M. R.; Yaghi O. M. Water Adsorption in Porous Metal–Organic Frameworks and Related Materials. J. Am. Chem. Soc. 2014, 136 (11), 4369–4381. 10.1021/ja500330a. 24588307

